# Tuberculosis in the Udder of the Milch Cow, and Its Effects upon the Milkz*

**Published:** 1885-04

**Authors:** B. Bang

**Affiliations:** Teacher at the Royal Veterinary School and Agricultural Academy, Copenhagen


### Art. XI.—TUBERCULOSIS IN THE UDDER OF THE MILCH COW, AND ITS EFFECTS UPON THE MILK.*

BY DR. B. BANG,
[Teacher at the Royal Veterinary School and Agricultural Academy,
Copenhagen.

My purpose in calling the attention of this honorable body to Tuberculosis in the Udder of the Milch Cow is with the hope of contributing somewhat to the knowledge of the Medical Profession with reference to the important relation existing between tuberculosis in the domestic animals and man. It has been acknowledged by all sides, that the danger of the transmission of this disease to man from animals, lies chiefly in the consumption of the milk from diseased cows, it being one of the chief articles of food among human beings, and especially because it is largely used in an uncooked condition. Although we must, *a priori*, assume that all milk derived from a cow complicated with tuberculosis, carries with it an infectious character, this is still greater when the organ of its production, the udder, is the seat of tuberculosis processes. Virchow, Bollinger, Galtier, Johne, Koch and others have all emphasized this idea. Galtier even asserts that the milk only has virulent properties when the udder is affected. Koch endorses this opinion. In his work upon the etiology of tuberculosis he says: "It is above all things necessary to infection, that the milk is infected with tubercle bacilli. This does not appear to be the case save when the milk glands are also the seat of the processes of tuberculosis." Further he says: "As the nodes of 'Perlsucht,' are but seldom met with in the udder, it is probable that the milk of such cows will be found frequently free from infectious properties. This fact explains the contradictory conclusions of different authors who have busied themselves in experimenting with the milk from such cows. Some report to have received positive results from such experiments, and their assertions are of such a nature that we cannot doubt the correctness of their observa-

*A paper read before the International Medical Congress at Copenhagen. Translated from advance sheets of the *Zeitschift für Thiermedicin*, Munich, with the kind consent of the Editor, Prof. Johne, of Dresden.

tions. Others, however, have only observed negative results as the conclusion of such experiments. Both parties have probably been correct. Positive results would only, then, follow in cases wherein the milk contained tubercle bacilli; negative with such as was free from them."

From these quotations it is evident that Koch is not inclined to look upon the consumption of the milk from cows afflicted with tuberculosis as of very great practical importance, as he does not consider that the tuberculosis of animals plays as important a role in the etiology of tuberculosis in man, as many other authors have done.

It will be my task to emphasize the importance of such milk as a source of infection. I hope to do so by proving the frequency of tuberculosis in the udder, as well as the highly virulent properties of the milk secreted by such udders.

It is well known that the processes of tuberculosis complicate the udder as well as the other organs, although we cannot say that veterinarians have given the attention to this particular part of the question which its importance deserves. As far as I know, its occurrence in the udder is most frequently mentioned in German literature.

Franck (Gubürtshülfe) describes the disease as follows: "The udder is frequently invaded by numerous tubercles of variable dimensions. The disease does not generally extend to the udder with, or shortly after parturition, but more frequently much later. The milk secretion diminishes, the milk itself becomes watery and viscid, then follows hypertrophy and induration of the affected part of the udder, or the whole organ, these conditions sometimes becoming very prominent, the organ weighing forty to fifty pounds; sometimes we can feel the nodes by palpitation. The hypertrophy constantly increases, while the milk secretion as constantly decreases to absolute cessation. The udder frequently becomes almost as hard as a stone. General phenomena, aside from those common to tuberculosis, fail. On slaughtering the animal, one finds the udder to be sclerotic and infected by tubercles in various stages of development. The lymph glands in the vicinity of the udder also become complicated, as a rule. Small abscesses are frequently formed. Treatment is useless." Ackerman, Fünfstuck, Meyer, Dinter, Hartenstein, Brauer, and others have also reported cases of udder tuberculosis. In 1868 Färstenberg—"Die Milchdrusen der Kuh"—considered the disease under the name of "Sarcomatosis." He did not look upon it as of very frequent occurrence. Pflug mentioned having observed it in 1877. Galtier and Walley mention its occurrence in France and England respectively.

It is thus evident that this peculiar complication of the udder in the milch cow is by no means unknown. Pathologists have considered it as of a most important occurrence. Notwithstanding these facts, the disease has never been subjected to intimate analysis or thorough study.

It has been my fortune to observe an uncommonly large number of cases during the last few years. In 1881 I saw two very marked cases in the cattle hospital of our school. In answer to my inquiries, veterinarians have sent me three tuberculous udders, as well as a cow afflicted with the disease in this organ. During the next year I had no occasion to observe the disease, but during the last winter the cases accumulated so rapidly that in the course of seven months, seven different cows were found thus diseased in seven different dairies in the city or its vicinity. A renewed appeal to the veterinarians was rewarded by fourteen new cases. Six of the same were discovered at the Copenhagen Cattle Mart. I was enabled to make an intra-vital as well as post-mortal examination of most of them.

An intra-vital diagnosis of this complication can be generally made without difficulty, and very early in the disease.

In brief, this complication is not characterized by any perceptible general disturbances. It frequently presents itself as a very diffuse, painless, intumescence of one—seldom two—quarters of the udder, most frequently one of the posterior ones. The tumefaction is generally considerable when the veterinarian's attention is first called to it. This is chiefly ascribable to the little importance which the dairyman frequently gives to such occurrences. From personal observation, I have, however, come to the conviction that no inconsiderable degree of tumefaction can occur in a very few days.

It is worthy of especial attention that such a markedly tumefied udder can at first yield an apparently healthy milk. This fact is of essential value in the differential diagnosis between this complication of the udder and those of a purely inflammatory character, whereby the milk secretion is always changed in its character, frequently to a very high degree.

In cases of violent mastitis a very acute tumefaction of the organ takes place in a few hours, accompanied by fever; in such cases either no milk at all is secreted, or only a thin, serous, sometimes offensive smelling fluid. In milder forms of inflammation the secretion may present a more milk-like appearance, but it is always thin, watery, and contains whitish, floculent, or caseous masses of variable dimensions.

Only when the inflammation has essentially subsided can an apparently normal milk be again secreted, though the intumescence may not have entirely disappeared. In violent mastitis the secretion becomes gradually purulent in character, abscesses being a frequent occurrence in the substance of the gland.

In cases where I meet with a very recent, diffuse, painless, and tolerably firm intumescence of the udder, or a portion of the same, and from the teat am enabled to press out a good appearing milk, I consider that I am warranted in diagnosing the trouble as Tuberculosis. In only one case have I erred in my diagnosis. In this one I found a moderate tumefaction of a quarter of the udder, of twelve days' duration—swollen condition of neighboring glands—milk but little changed, somewhat more watery than normal. In the course of three months the condition of the milk had shown no further change, but the complicated portion of the udder had gradually become smaller than the other quarters. The cow remained well. In all other cases the complicated quarter of the udder had gradually increased in size and density, until finally it became almost as hard as a stone and of an immense size, while the non-complicated parts as gradually atrophied. During the course of the disease the neighboring parts were frequently the seat of extensive œdematous tumefaction, which gradually diminished, and, according to my experience, never began with the severity which generally accompanies severe inflammatory processes in the udder. The secretion of the lacteal glands generally retained its milky appearance for months, becoming gradually more watery in character, to become finally somewhat flocculent and serous, but the secretion continued. If one first meets the disease in this stadium it can be easily mistaken for mammitis. If, however, we take into consideration the *very important intumescence of the udder*, as well as its unnatural hardness, *and the complete failure of suppuration*, a mistaken diagnosis is not easily made. The diagnosis of tuberculosis is essentially supported by the tumefaction of the adjoining lymph glands, which often become very much enlarged. When the anterior quarters are alone complicated these glands are not found to be swollen. In all such cases one should be very careful in obtaining the anamnesis, *i. e.*, the history of the case. If one finds that the tumefaction has gradually developed, and that the secretion appears comparatively normal, there is no doubt with regard to the diagnosis.

These conditions do not seem to have received the attention which their importance demands. That the diseased portions of the udder secretes an apparently good looking milk during the first stages of the complication is of manifest diagnostic importance. It is self-evident that such milk will be used for human consumption so long as it appears to be normal in character. I could quote sufficient proof that such is the case. It shows the necessity of the most exact control of the milch cows by competent veterinary inspectors; for such milk is frequently mixed with other, which thereby contaminates and makes the whole product of the dairy dangerous to the consumers.

As to the infectiousness of milk secreted by such portions of the udder of the cow as are the seat of tuberculous processes, I must say that I have always been able to demonstrate the presence of a great number of the bacilli of tuberculosis. It is not always so easy, however, to find the bacilli; when present in small numbers one may have to examine many preparations before finding them. In other cases they are easily to be found.*

---

* We have never had opportunity to examine such milk, but judging from the analogy, and from the examination of human sputum, I should say that they would be found in the flocculent material, which is to be demonstrated by spreading the same thinly on a covering glass and allowing it to dry, and then coloring it with fuchsin or gentian violet by methods which will be described in the Journal, as they have already been in last year's editions.—EDITORS.

In one case I was enabled to discover over two hundred bacilli in one covering glass specimen of such milk, although the milk appeared comparatively normal one month after the intumescence of the gland had appeared. In the beginning they are not generally so well represented, but when we find several on each covering glass it is safe to assume that in drinking a glass of such milk a person would introduce into his system millions of these disease-producing germs. I must here remark that the bacilli generally contain spores, which greatly increases their virulent qualities. Koch has asserted that, like those of anthrax, only the bacilli which contain spores are capable of causing infection and withstand the action of the juices of the stomach.

When the milk from such a portion of the udder is found in this condition there is every hygienic reason for condemning the whole yield of the cow, for I have always found the milk to possess highly virulent characteristics also.

My attention was called to this important point very early in my studies of the subject. I found that the disease extended to the other portions of the udder very early in the malady, so that it is probable that the milk drawn from the anterior cisterns, notwithstanding its normal appearance, is already afflicted with virulent qualities. I also found in such cases that small miliary nodes had already made their appearance in the walls of the milk cisterns. As early as 1882 I was enabled to produce infection in rabbits by the subcutaneous injection of small quantities of such milk. The animals first demonstrated the phenomena of disease in the course of two months, and died in from 2½ to 3½ months of tuberculosis. I have since repeated these experiments with the most successful results. I used the milk from the diseased as well as the apparently healthy parts of the udder; in each case tuberculosis was the positive result. In each case I subjected two rabbits to the experiment, injecting about four grammes of the milk into the abdominal cavity. Numerous other experiments of a similar nature were followed by like positive results in the course of from two to three months. As to the virulence of the milk from the apparently healthy portions of the udder, it is well to remark that my experiments have been confirmed by those of May.

These experiments should sufficiently demonstrate the fact, that the milk from the apparently non-complicated portions of the udder contains equally virulent elements with the parts which ocular and palpitatory examinations demonstrates to be the seat of tuberculous processes.

It is evident that all dairies should be subjected to veterinary inspection, and that all cows in which tuberculous processes are diagnosed in the udder should be at once slaughtered.

I must here especially call attention to the fact that I have frequently found unquestionable cases of tuberculous mammitis in cows that were otherwise apparently healthy, which leads me to assume that tuberculosis can occur in the udder of the cow as a primary complication. I have, however, found that in such cases the bronchial or other glands were frequently the seat of old caseous or calcified processes which, in all probability, had connection with processes in the lacteal glands. Whether there are other means of infection by which the bacilli can gain direct access to the milk glands is still an open question.

Notwithstanding the assertions of Koch, it is certain that cows frequently eject a form of sputum from the mouth, and as the intestinal canal is also the seat of tuberculous ulceration, it is also probable that tubercle bacilli are passed off with the fæces. The uterus mucosa has also been found to be the seat of extensive tuberculosis, and I have myself found tubercle bacilli in the vaginal discharges from such cows. Tuberculous abscesses also occur in the retro-pharyngeal regions, and I have found bacilli in their contents. That consumptive attendants may be the cause of the infection of cows and other animals is not without most positive evidence.

It is certainly of theoretical, as well as practical interest, to know if the udder can be directly infected by tuberculous elements from without, *i. e.*, through the canals in the teats. That the disease can and does occur as a primary complication of the udder and that general tuberculosis may then result, has been proved beyond all question by my observations.

In those cases where tuberculosis of the udder followed as a secondary complication, I have found that pulmonary complications were generally anticipatory; in other cases, tuberculosis of the uterus or fallopian tubes.

The pathological anatomy of tuberculosis of the last named organs deserves some consideration on our part. In the writings of authors we generally find it mentioned that one meets nodes in the uterus, and that tubercle in the udder, of varying dimensions, have also been seen. According to my ideas this description is very unsatisfactory. I have always found the affection to be very recent and diffuse, so that the complicated portions of the udder was the seat of extended intumescence. In saying this I do not mean to assert that all the swollen part possessed the same degree of hardness, but these tumefactions were not so sharply circumscribed as to warrant my designating them as nodular in character. In cases, where the course of the disease was of a very chronic character, the tumefactions were more sharply circumscribed. In others I have found neoplasmata of the size of a walnut or hen's egg in parts of the uterus which were not otherwise complicated.

If we make a section of that portion of the udder which is the seat of tuberculosis processes, we obtain a somewhat different picture, according to the duration of the disturbance. In all cases we find a firm intumescence of the complicated parts, the entire cut surface having a singularly homogeneous appearance. The tumefied portions are distinguished from the healthy by a sharply marked line of demarcation. In the earlier stages of the disease, *i. e.*, the first few weeks, one finds the lobuli of the glands swollen, moist, and of a homogeneous grayish color. By more exact inspections we are enabled to see somewhat harder yellowish objects in the substance which evidently correspond to the acini of the gland, and besides these branching yellowish striæ which corresponded to the smallest lacteal ducts. The larger ducts are filled with a yellowish caseous mass, or their walls are coated with the same. This caseous mass will be found to be rich in bacilli. Nodulation in the walls of the larger ducts and milk-cisterns can also be seen.

The tissues that are most intensely swollen have a variegated reddish color that is due to circumscribed extravasations of blood. The veins of the udder are the seat of extensive thrombosis. The tuberculous nature of the disease is not always distinctly marked by the microscopic examination of such fresh cases. One could easily mistake such conditions for the result of a simple chronic inflammation. The true nature of the complication would, however, manifest itself to the competent observer by the caseous affection of the smaller lacteal ducts as well as the tumefied condition of the neighboring lymph glands, which are generally the seat of exquisite tuberculous infiltration.

The phenomena rapidly changes, however, with the progress of the disease. The caseous degeneration of the lobuli of the glands extends rapidly, forming dry, yellowish nodes of an irregular form, which in some places occupy only a portion of the lobulus, while in others they complicate the whole of this portion of the gland. Sometimes several of the lobuli coalesce to a caseous mass. Sometimes the lacteal ducts are found to be distended—ectosis—and filled with a caseous secretion. Interstitial processes are to be seen in and around the lobuli, as well as the complicated lacteal gland. Sometimes the interstitial processes predominate, single neoplasms being only seen here and there in the indented substance of the gland. In very few cases I have met with a soft or fluid contents in the lobuli.

Tubercle bacilli were to be found in the caseous products, as well as in the accumulations of round cells, and in the neoplastic connective tissues.

In my description of the symptoms of udder tuberculosis I have mentioned some experimental proofs of the same, with reference to the milk.

Inoculation experiments can only prove that the milk used contained virulent, *i. e.*, infectious elements. Feeding experiments can only show whether the tuberculosis of the udder is capable of being transmitted from the cow to other animals, consequently to human beings. Such experiments have been frequently made, with many contradictory results. According to the results collected by Johne of ninety-one such experiments made with the milk from tuberculous cows, 30 per cent. gave positive, and 59 per cent. negative results.

I have already mentioned that many observers have tried to explain this variation in the results, by the assumption that the udder could only have been complicated in the cases followed by positive results. I cannot agree with this assumption, as tuberculosis of the udder is such a well-marked complication that it appears improbable that its diagnosis could escape the attention of any competent observer. I shall endeavor to justify this remark later on. The question is of such vital interest that the repetition of feeding experiments with the milk from cows that were unquestionably afflicted with udder tuberculosis is warranted.

I have, therefore, fed five young pigs and three rabbits with the milk from two different cows. Both experiments had to come to an earlier termination than I desired, on account of the cessation of the milk secretion and death of the cow. The animals were, however, fed almost exclusively with said milk for two weeks, and later the milk obtained was mixed with the food.

From the first cow I only used the apparently normal milk from the seemingly healthy quarters of the udder; from the second cow I used the secretion from all parts of the udder.

Two pigs and one rabbit were fed with the milk from the first cow. The pigs received the milk from the 1st of January until the 3d of February, 1884. They were killed on the 1st of August, and in both the mesentery glands were the seat of caseous degeneration, as well as those in other parts of the body. Caseous ulceration in the vicinity of the valvula Bouhini in both; tubercle in the lungs, liver and spleen of each animal.

The rabbit was killed on the 29th of July. It had not shown any symptoms of importance. It was tolerably fat, but well-developed tuberculosis was found; ulceration in the intestines, as in the swine; also tubercles in the intestinal cavities, calcified nodes in the mesenteric glands, and isolated tubercles in the kidneys and lungs.

As was to be expected, the tubercular processes were much more manifest in the animals fed with the milk from the second cow. Two rabbits and three pigs were used in this case. The period of experimental feeding extended from the 8th of May to the 14th of June. The animals were killed on the 8th of August. The rabbits had not thrived well during the last part of the time; in both there was extensive tuberculosis of the intestinal parieties; extensive ulceration in the apex of the cœcum, and numerous caseous follicular ulceration dispersed through the intestines, as well as extensive ulceration in the vicinity of the valvula Bouhini; caseous degeneration of the lymph glands and tuberculous infiltration in the liver. Lungs, spleen and kidneys apparently healthy.

The pigs were apparently healthy intra vitam. In all of them, however, caseous nodes were to be seen in the lymph glands of various parts of the body. In two, caseous follicular ulceration in the vicinity of the valvula Bouhini, and in one of these pigs, and the third, tuberculosis pulmonum.

In the caseous nodes of the mesentery glands as well as in the caseous pus of the follicles in the intestines, I found tubercle bacilli, but never in great numbers.

The importance of these experiments from the hygienic point of view as well as the economical to the consumer in the first place, and the breeder of animals in the other, cannot be overestimated.

Dr. L. Anderson of Seeland, reported to me a case of the transmission of tuberculosis to a calf from a cow having udder tuberculosis by means of natural feeding. The lady of the house who had been previously considered healthy, though perhaps complicated somewhat with hereditary tendencies, commenced to cough at the same time and ended with a rapidly developing phthisis. During the early stage of her disease she gave birth to a child which was fed with the milk from one cow, also tuberculous, and which died from the disease within a year. Within six months the child also died of tuberculosis. Although the connections are somewhat complicated, it is very probable that the child derived its disease from the cow's milk.

As to the question whether the milk contains infectious elements if the udder is tuberculous or not, there is some difference of opinion among authors. May's experiments seem to prove that it is. I have made experiments with the milk from two cows that had very marked tuberculosis, but not of the udder. The inoculation by one rabbit gave a negative result, while by the other they were of a most decided positive nature. I must therefore differ from Koch in concluding that tuberculosis can be generated from the milch cows having the disease, even when no complications can be found in the udder. In one such case I have been able to find bacilli in the milk, though I could find no evidence of disease in the udder. I must mention some experiments made with milk that had been treated by the centrifugal method, which is used to separate the cream. One advantage of this method is that the milk is thereby freed from dirt and foreign elements, which accumulate on the sides of the machine. An interesting question, therefore, arose in my mind—if the tubercle bacilli were also thus separated from the milk? Four rabbits were each inoculated, two with the clear milk, and two with the sediment. At the same time I made covering glass preparations from both materials. But very few bacilli could be found in the milk itself, while large numbers were to be seen in the sediment. They seemed to be mostly in the body of the lymph-cells. In one cell from the sediment I could count twenty bacilli. This examination demonstrated that the centrifugal treatment of milk from cows with udder tuberculosis is insufficient to separatethe bacilli securely from the fluid, though large numbers are undoubtedly precipitated on the sides of the machine.

The result of the inoculations was as follows:

In the first series, one of the rabbits treated with sediment material was soon attacked with septic phlegmonia-cellulitis, and died in twenty-three days. No microscopic appearance of tuberculosis. The other rabbit died fourteen days later from a most acute miliary tuberculosis, which was accompanied by inflammatory exudation in the abdominal cavity. In the other series, the animals in which sediment was inoculated, both died of miliary tuberculosis.

Of the four rabbits in which the skimmed milk was inoculated, one from each series was killed on the 9th of August. Both were infected with tubercles, but were not so emaciated as those that had received sediment material.

The third rabbit, which was inoculated with the milk on the 17th of June, died of general tuberculosis the 29th of August; the fourth on the 5th of September. The tuberculous neoplasmata in the organs of these animals had obtained considerable size. The centrifugal treatment is not sufficient to rob the milk from cows with tuberculosis of the udder of its virulent qualities.

EXPERIMENTS WITH REFERENCE TO THE INFLUENCE OF HEAT UPON MILK CONTAINING TUBERCULOSIS BACILLI.

May and others have shown that, when such milk is cooked to the boiling point, it loses its virulence, *i. e.*, the bacilli are killed. We decided upon a temperature of 72°, because experiment has shown that when it is heated to 60°–70° C., the milk retains its good qualities as well as when cooked, without changing its taste, and it might be expected that milk dealers would soon ascertain this fact. We then took the sediment from the centifugal machine, which was rich in bacilli, and warmed it for a quarter of an hour in a warm bath heated up to 72° C. One gramme of this material was subcutaneously injected into two rabbits. Both were killed at the expiration of eight weeks, and, aside from some small gangrenous nodes in the liver, were healthy.

Herr v. Storch, superintendent of our chemical laboratorium, subjected the milk from a cow with udder tuberculosis to chemical analysis, with the following results:

"When we compare the two analyses of the milk taken from the diseased gland with one another, we find that the percentage of fat and milk-sugar had materially diminished between the 7th of May and 6th of June; it is striking that the milk-sugar should have almost entirely disappeared. The milk from the apparently healthy, non-tumefied portions of the udder was rich in fat and albuminoids; in its other chemical constituents it varied but little from normal cow's milk.

"The appearance of the serum of the milk from the diseased portion of the gland caused me to analyze the ascher from this milk, and that from the apparently healthy portions of the udder. The same demonstrated striking variations on the part of the milk from the diseased quarter. The proportion of lime and phosphate was much less, with the soda salts greatly augmented. The soda salts were also much more profusely represented in the milk from the healthy portion of the udder than in normal milk; the other salts were about normal in quantity.

"I was singularly struck with the almost total absence of chlorides in the milk from both portions of the udder of this cow; but as I have met with this condition in normal milk it probably had nothing to do with the disease."

